# Dataset of the molecular dynamics simulations of bilayers consisting of short amyloidogenic peptide VDSWNVLVAG from Bgl2p–glucantransferase of *S. cerevisiae* cell wall

**DOI:** 10.1016/j.dib.2016.09.043

**Published:** 2016-10-03

**Authors:** Anna V. Glyakina, Nikolai K. Balabaev, Oxana V. Galzitskaya

**Affiliations:** a Institute of Protein Research, Russian Academy of Sciences, 142290 Pushchino, Moscow Region, Russia; b Institute of Mathematical Problems of Biology RAS, Keldysh Institute of Applied Mathematics of Russian Academy of Sciences, 142290 Pushchino, Moscow Region, Russia

**Keywords:** Amyloidogenic peptide, Amyloid fibrils, Bilayers, MD simulation, Stability, Beta-strands

## Abstract

The amyloidogenic peptide V**D**SWNVLVAG from Bgl2p–glucantransferase of *Saccharomyces cerevisiae* cell wall and its modifying analog V**E**SWNVLVAG were taken for the construction of four types of bilayers which differ by orientation of the peptides in the layers and of the layers relative to each other. These bilayers were used as starting models for the molecular dynamics (MD) at three charge states (neutral, pH3, and pH5). The changes of the fraction of secondary structure during 1 ns simulations were received for 96 MD trajectories. The data article contains the necessary information for the construction of models of β-strands organization in the oligomer structure. These results were used in the associated research article “Structural model of amyloid fibrils for amyloidogenic peptide from Bgl2p–glucantransferase of *S. cerevisiae* cell wall and its modifying analog. New morphology of amyloid fibrils” (Selivanova et al., 2016) [1].

**Specifications Table**

**Table:** 

Subject area	*Biophysics*
More specific subject area	*Molecular dynamics simulations of short peptides*
Type of data	*Table, figure*
How data was acquired	*Software for molecular dynamics simulation and data processing (PUMA, YASARA)*
Data format	*Analyzed*
Experimental factors	*Using template for construction of initial structures for MD simulations*
Experimental features	*Temperature of simulation* 27 °C, *pH 3, 5*
Data source location	*Institute of Mathematical Problems of Biology RAS, Keldysh Institute of Applied Mathematics of Russian Academy of Sciences, 142290 Pushchino, Moscow Region, Russian Federation*
Data accessibility	*Data is within this article*

**Value of the data**

• The data allows others to determine the most stable packing of peptides in bilayers.
• The studies of short peptides, which are capable to form amyloids are important because it provides the additional information for other researches in this field of study.
• Technical information describing the procedure of construction of the bilayers with the given amino acid sequences may be useful.

## 1 Data

**Fig. 1:**
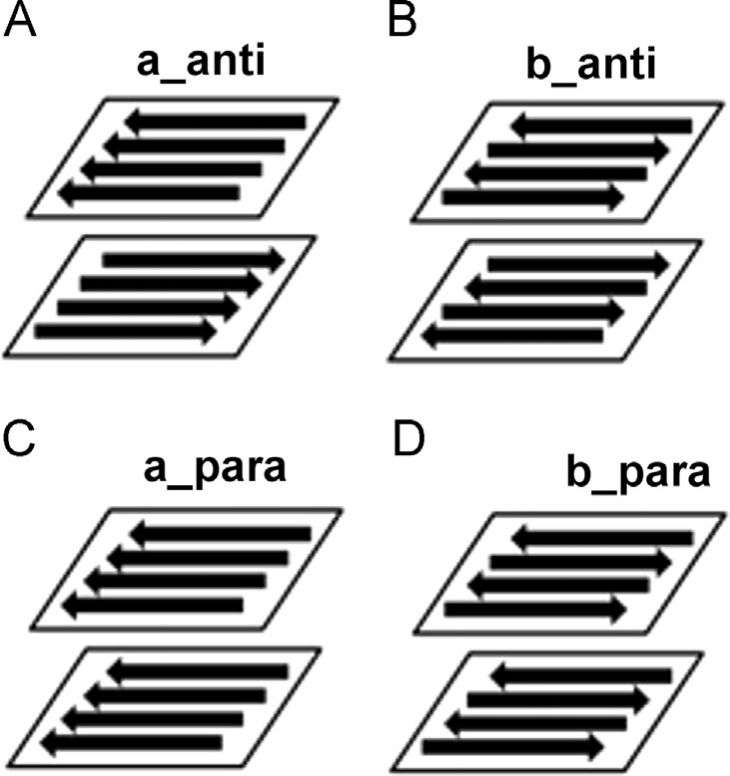
Possible variants of orientation of the peptides in the layers and of the layers relative to each other.

**Fig. 2:**
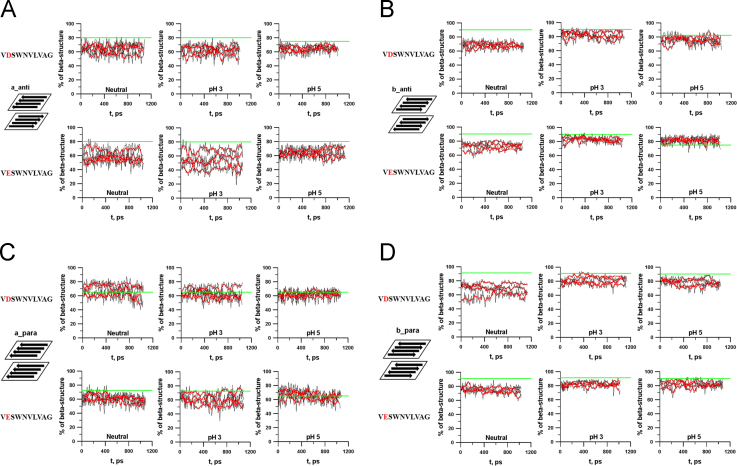
Change of the fraction of secondary structure in the bilayers during the simulation: a_anti (A), b_anti (B), a_para (C) and b_para (D). There are four curves for each bilayer. For better representation all graphs were averaged over 50 ps (red curves). The straight green line indicates the level of β-structure in the bilayers before the simulation.

The amino acid sequence V**D**SWNVLVAG corresponds to fragment 166–175 of protein glucantransferase Bgl2p from the yeast cell wall [2] which is enable to form amyloids and its modifying analog V**E**SWNVLVAG were taken for the construction of the bilayers [1]. Possible variants of orientation of the peptides in the layers and of the layers relative to each other are presented in Fig. 1. Simulations of bilayers were done at three charge states: neutral, pH3 and pH5. The fraction of secondary structure in each bilayer before, during and after the simulation was calculated using the YASARA program [3] and the results are represented in Table 1, Table 2 and Fig. 2.

## 2 Experimental design, materials and methods

### 2.1 Construction of the bilayers

Structures 3N3E (zebrafish αA crystallin) and 2MVX (amyloid-β fibrils Aβ(1–40)) from Protein Data Bank were taken for the construction of the bilayers. Fragments from amino acid residue 95 to 104 (chain A), and from amino acid residue 110 to 119 (chain B) were taken from structure 3N3E. Fragments from amino acid residue 10 to 19 (chain A, B, C, D) were taken from structure 2MVX. All these fragments correspond to β-structure. Then, using the YASARA program [3], the following amino acid sequences V**D**SWNVLVAG and V**E**SWNVLVAG were fitted in each of these fragments. Structure 3N3E was the template for the construction of the bilayers b_anti and b_para (Fig. 1B and D). Structure 2MVX was the template for the construction of the bilayers a_anti and a_para (Fig. 1A and C).

Thus, β-layers in which β-strands were arranged parallel or antiparallel relative to each other were obtained. Then, the first β-layer and the second β-layer were arranged parallel or antiparallel to each other at a distance of 10 Å.

For each bilayer shown in Fig. 1 three charge states were considered (Table 3):

• neutral system;
• system corresponding to pH3 (the *N*-terminus was positively charged);
• system corresponding to pH5 (the *N*-terminus was positively charged and the *C*-terminus and Asp (or Glu) were negatively charged).

### 2.2 Simulation protocols

**Fig. 3:**
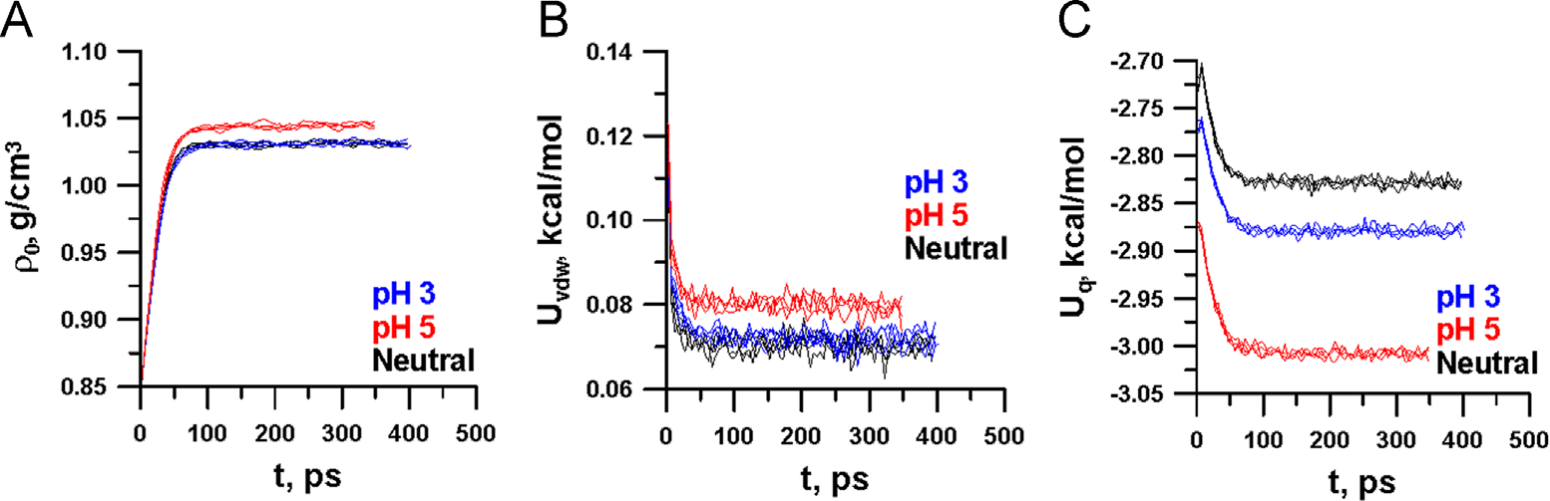
Dependences of the densities (A), van der Waals (B) and Coulomb (C) energies on the relaxation time for the bilayer b_para GluNB at three charge states: neutral (black curves), pH3 (blue curves) and pH5 (red curves). There are four curves for the bilayer in each charge state.

Each bilayer was surrounded by more than 1500 water molecules. Molecular dynamics simulations were performed using the program PUMA [4]. Initially, relaxation of the bilayers in the NPT ensemble for 400 ps was performed under periodic boundary conditions using the AMBER99 force field [5]. The TIP3P model of water [6] was used. The constant pressure and temperature were maintained by a Berendsen barostat [7] and a collisional thermostat [8], [9]. For every system, four independent simulations were done. During the relaxation the energies (van der Waals and Coulomb) of the systems begin to fluctuate around certain equilibrium values and their densities have reached equilibrium values (Fig. 3). The following simulation for 1 ns was done in the NVT ensemble.

## Figures and Tables

**Table 1:** Fraction of β-structure (%) in the bilayers before the simulations.

Amino acid sequence	Type of the system	a_anti	b_anti	a_para	b_para
V**D**SWNVLVAG	1) Neutral	80	90	65	91
	2) pH3	80	90	65	91
	3) pH5	75	83	65	90
V**E**SWNVLVAG	1) Neutral	80	90	73	91
	2) pH3	80	90	73	91
	3) pH5	80	75	65	90

**Table 2:** Fraction of β-structure (%) in the bilayers after the simulations.

Amino acid sequence	Type of the system	a_anti	b_anti	a_para	b_para
V**D**SWNVLVAG	1) Neutral	65±3	68±1	64±5	67±4
	2) pH3	63±4	**81±3**	62±4	**81±3**
	3) pH5	65±1	**75±3**	64±2	**76±1**
V**E**SWNVLVAG	1) Neutral	58±5	**72±1**	57±2	**73±3**
	2) pH3	53±7	**82±1**	60±4	**82±2**
	3) pH5	65±4	**82±1**	63±3	**82±1**

**Table 3:** Distribution of charges in the systems.

Type of system	Charge			
	The total charge of the system (8 peptides)	*N*-terminus	AspNB or GluNB	*C*-terminus
1) Neutral	0	0	0	0
2) pH 3	+8	+	0	0
3) pH 5	−8	+	–	–
